# Neuroprotective Effects of Bone Marrow Mesenchymal Stem Cells on Bilateral Common Carotid Arteries Occlusion Model of Cerebral Ischemia in Rat

**DOI:** 10.1155/2016/2964712

**Published:** 2016-10-25

**Authors:** Bagher Pourheydar, Sara Soleimani Asl, Mostafa Azimzadeh, Adel Rezaei Moghadam, Asghar Marzban, Mehdi Mehdizadeh

**Affiliations:** ^1^Department of Anatomical Sciences, Faculty of Medicine, Urmia University of Medical Sciences, Urmia, Iran; ^2^Neurophysiology Research Center, Faculty of Medicine, Urmia University of Medical Sciences, Urmia, Iran; ^3^Anatomy Department, School of Medicine, Hamadan University of Medical Sciences, Hamadan, Iran; ^4^Cell Therapy Division of Endometrium and Endometriosis Research Center, Hamadan University of Medical Sciences, Hamadan, Iran; ^5^Young Researchers and Elite Club, Islamic Azad University, Yazd Branch, Yazd, Iran; ^6^Faculty of Veterinary Medicine, Islamic Azad University, Tabriz Branch, Tabriz, Iran; ^7^Department of Pediatrics, School of Medicine, Zanjan University of Medical Sciences, Zanjan, Iran; ^8^Cellular and Molecular Research Center, Department of Anatomy, Faculty of Advanced Technologies in Medicine, Iran University of Medical Sciences, Tehran, Iran

## Abstract

Cell therapy is the most advanced treatment of the cerebral ischemia, nowadays. Herein, we discuss the neuroprotective effects of bone marrow mesenchymal stem cells (BMSCs) on rat hippocampal cells following intravenous injection of these cells in an ischemia-reperfusion model. Adult male Wistar rats were divided into 5 groups: control, sham (surgery without blockage of common carotid arteries), ischemia (common carotid arteries were blocked for 30 min prior to reperfusion), vehicle (7 days after ischemia PBS was injected via the tail vein), and treatment (injections of BMSC into the tail veins 7 days after ischemia). We performed neuromuscular and vestibulomotor function tests to assess behavioral function and, finally, brains were subjected to hematoxylin and eosin (H&E), anti-Brdu immunohistochemistry, and TUNEL staining. The ischemia group had severe apoptosis. The group treated with BMSCs had a lower mortality rate and also had significant improvement in functional recovery (*P* < 0.001). Ischemia-reperfusion for 30 min causes damage and extensive neuronal death in the hippocampus, especially in CA1 and CA3 regions, leading to several functional and neurological deficits. In conclusion, intravenous injection of BMSCs can significantly decrease the number of apoptotic neurons and significantly improve functional recovery, which may be a beneficial treatment method for ischemic injuries.

## 1. Introduction

Worldwide, cerebral ischemia is one of the leading causes of long-term disability, morbidity, and death [1, 2]. Reperfusion following cerebral ischemia induces neuroinflammation and excessive production of reactive oxygen species (ROS) [3, 4]. Under physiological conditions a homeostatic balance between the formation of oxygen-free radicals and their removal by endogenous scavengers exists [5]. During cerebral ischemia, reduction of glucose and oxygen transport to the brain leads to the generation of free radicals which damage lipids, DNA, and proteins, in addition to inflammation and breakdown of the blood-brain barrier (BBB), resulting in cell death [6–8].

Cerebral ischemia can lead to sensory, motor, cognition, and spatial learning disorders depending upon the location of the ischemic event [9–11]. Motor disorders associated with cerebral ischemia lead to disabilities that affect quality of life [12]. The hippocampus is one of the first areas of the brain affected by neurodegenerative diseases and injuries attributed to cerebral ischemia. The pyramidal neurons of the CA1 area of the hippocampus are the most sensitive neurons to hypoxia and subsequent death during ischemic conditions [13–15].

In animal models, it has been proved that the ischemia has injury mechanisms, including excitotoxicity, mitochondrial dysfunction, and oxidative stress. On the way to protect cells from cerebral ischemia, molecular chaperones or stress proteins and some antiapoptotic members of the BCL2 family of apoptosis regulatory proteins can protect mitochondrial function, reducing oxidative stress [16–18].

Currently, only a few effective clinical therapies exist for cerebral ischemia that lead to complete functional recovery [19]. Recently, stem cell-based therapy has provided a therapeutic tool for tissue repair and functional recovery in neurological diseases and cerebral ischemia [20–22]. Stem cells have the capacity of unlimited self-renewal and give rise to differentiated cells from various cell lineages [23–25]. They are classified according to origin as embryonic, fetal, or adult stem cells. Embryonic stem cells (ESCs) have restricted availability and form teratomas after transplantation. Due to ethical concerns, their application is limited [26, 27]. Among the stem cells, bone marrow mesenchymal stem cells (BMSCs) have greater potential use in the treatment of neurological disorders. These cells can be easily obtained from patients without ethical or immunological problems and can be produced in large numbers under in vitro conditions [28, 29].

Several studies have suggested that BMSCs can migrate to the injury site in the brain and differentiate into neurons and glial cells [30]. Previous studies have mainly focused on molecular and histological aspects of cerebral ischemia, rather than behavioral consequences. However, behavioral tasks are suitable tools for investigating the consequences of cerebral ischemia. The present study investigates the histopathological and behavioral effects of intravenously transplanted BMSCs in a rat experimental model of cerebral ischemia-reperfusion.

## 2. Methods and Materials

### 2.1. Animals

Adult male Wistar rats (*n* = 40) that weighed 250–300 g were obtained from the Animal House of the Faculty of Medicine at Urmia University of Medical Sciences, Urmia, Iran. Animals were maintained at 21 ± 1°C (50 ± 10% humidity) on a 12 h light/12 h dark cycle with access to water and food ad libitum. Animal care and the general protocols for animal use were approved by the Animal Ethics Community at Urmia University of Medical Sciences.

### 2.2. Experimental Design

We randomly divided the rats into 5 groups (*n* = 8) as follows: (1) control (intact) where the animals underwent no ischemia or treatment; (2) sham in which the animals underwent surgery without blockage of the common carotid arteries; (3) ischemia in which bilateral common carotid arteries were blocked for 30 minutes in order to induce ischemia; (4) vehicle in which the rats received 30 *μ*L phosphate-buffered saline (PBS) injected into their tail veins 7 days after ischemia; and (5) treatment in which the animals received BMSCs (1 × 10^6^ in a 30 *μ*L suspension) injected into their tail veins 7 days after ischemia.

### 2.3. Experimental Model of Cerebral Ischemia-Reperfusion

We used a modified method of Jingtao et al. [31] to induce cerebral ischemia-reperfusion. The animals were anesthetized with ketamine (80 mg/kg, Daroopakhsh, Iran) and xylazine (10 mg/kg, Daroopakhsh, Iran). The rats were then placed in the supine position on an operating table and covered with a warming blanket. Each animal's body temperature was controlled with a temperature control unit during surgery.

After shaving and prepping with Betadine, a midline incision was made that exposed both common carotid arteries. Dissection was made between sternocleidomastoid and sternohyoid muscles parallel to the trachea. Each common carotid artery was freed from its adventitial sheath and carefully separated from the vagus nerve. For induction of ischemia, both common carotid arteries were occluded using microaneurysm clamps for 30 min followed by reperfusion. The skin was stitched with silk suture.

### 2.4. Isolation and Culture of Bone Marrow Mesenchymal Stem Cells (BMSCs)

Bone marrow was isolated in sterile conditions from 8-week-old male Sprague Dawley rats (weights: 250–300 g) as described in detail by Azizi et al. [32]. Briefly the rats were given an overdose of pentobarbital after which their tibia and femurs were excised. Both ends of each bone were cut and the marrow was aspirated with 5 mL DMEM (Sigma-Aldrich) and a 25-gauge needle. The resultant suspension was centrifuged at 800 rpm for 5 min and the supernatant was removed. Subsequently, the marrow cells were suspended in 10 mL of DMEM supplemented with 10% fetal bovine serum (FBS), 2 mL glutamine, 100 U/mL penicillin (Sigma-Aldrich), and 100 U/mL streptomycin (Sigma-Aldrich).

**Figure 1:**
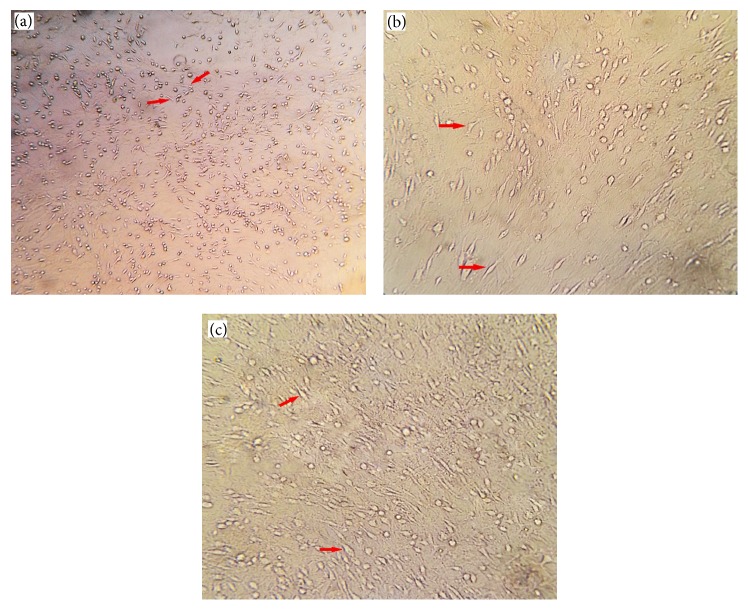
Various stages of cultured bone marrow stromal cells. Bone marrow stromal cell morphology in the early (a), middle (b), and late (c) subculture stages.

After 48 h, the nonadherent BMSCs were removed by replacing the medium. When 80% confluency was reached, cells were detached and harvested after a 5 min exposure to 0.25% trypsin/1 mM EDTA (Sigma-Aldrich, USA) at 37°C. Cells were subsequently passaged into four subcultures for further incubation. Various stages of cultured bone marrow mesenchymal stem cells (BMSCs) have been shown in Figure 1.

### 2.5. Labeling of BMSCs and Cell Transplantation Procedure

The cells were labeled with a 3 *μ*g/mL bromodeoxyuridine (Brdu) solution added to the incubation medium 72 h prior to transplantation [33].

### 2.6. Cell Transplantation Procedure

The 8 animals in group 5 (treatment) were anesthetized with ketamine (80 mg/kg) and xylazine (10 mg/kg) at 7 days after ischemia. Approximately 1.0 × 10^6^passage 3 BMSCs suspended in 30 *μ*L PBS were intravenously given into the rats' tail veins over a 5-min period. The vehicle group only received an infusion of 1 mL PBS injected into their tail veins. Immunosuppressant was not used for any animal in this study.

### 2.7. Immunohistochemical Analyses

At 12 days after ischemia, the rats were deeply anesthetized with sodium pentobarbital (100 mg/kg, IP) and then transcardially perfused with 4% paraformaldehyde in 0.1 M phosphate buffer (pH 7.3). After transcranial perfusion, the animals' brains were removed and postfixed in 4% paraformaldehyde for one week. Next, we processed the tissues and a series of adjacent 5 *μ*m thick sections were cut from paraffin blocks in the coronal plane. Immunohistochemical staining was used to detect the distribution of transplanted BMSCs in the hippocampus, as follows: deparaffinization of samples in xylol (Merck, Germany), hydration in a graded alcohol series (100, 96, 80, and 70%), incubation in 50% formamide (Merck, Germany), incubation in 2x standard sodium citrate (SSC, Merck, Germany) for 2 h at 65°C, incubation in 2 N HCl (Merck, Germany) for 30 min at 37°C, rinse in 0.1 N boric acid (Merck, Germany, pH 8.5) for 10 min, followed by a PBS wash, incubation with mouse anti-Brdu antibody (Sigma, Germany) overnight at 4°C, rinse in PBS (3 times for 10 min), incubation with secondary antibody conjugated with horse radish peroxidase (goat anti-mouse IgG) (Sigma, Germany) for 2 h, and incubation with diaminobenzidine tetrachloride hydrate (DAB, Sigma, Germany) for 5 min, after which the slides were counterstained with hematoxylin, mounted, and inspected under a light microscope.

### 2.8. Terminal Deoxynucleotidyl Transferase-Mediated dUTP end Labeling (TUNEL)

Apoptosis was determined by terminal deoxynucleotidyl transferase-mediated dUTP end labeling (TUNEL) which detects the DNA fragments of apoptotic cells. TUNEL staining was performed with the In Situ Cell Death Detection Kit (Roche Molecular Chemical, cat. number 11684817910). Briefly, the sections were dewaxed and rehydrated in xylol (Sigma, Germany) and a graded series of 100%, 90%, 80%, and 70% ethanol. Endogenous peroxidase activity was blocked by incubating with 0.3% H_2_O_2 _in methanol for 30 min. For permeabilization of tissue, the sections were incubated with 20 *μ*g/mL proteinase K (Roche, Germany) in 10 mM Tris HCl (pH 7.5) for 10 min at room temperature. Then, the sections were incubated in 50 *μ*L of TUNEL solution (In Situ Cell Death Detection Kit, Roche, Germany) for 1 h at 37°C. After incubation with converter POD, HRP for 1 h at 37°C, the sections were incubated with DAB (Sigma, Germany) chromogen at 37°C. Cells were counterstained with hematoxylin (Sigma, Germany) for 20 s. Sections were dehydrated in a graded series of 70%, 80%, 90%, and 100% ethanol and cleared in xylol for 15 min. After mounting the sections were examined by a light microscope.

### 2.9. Cell Counting and Apoptotic Index Determination

We counted the numbers of apoptotic cells by randomly selecting 20 microscopic fields in the hippocampal CA1 and CA3 areas. Apoptotic pyramidal neurons were counted at 40x magnification after which the mean number of apoptotic cells in the CA1 and CA3 areas was calculated. The total numbers of cells in each field were counted and the mean number was calculated. We determined the apoptotic index for each group as follows:

(1) $$\begin{array}{c} \text{Apoptotic index}=\frac{\text{Number of apoptotic cells}}{\text{total number of cells}}\times 100. \end{array}$$

### 2.10. Histopathological Assessment

A number of the 5-*μ*m prepared sections were used for histopathological assessment. Hematoxylin and eosin (H&E) stained sections were studied under a light microscope (400x) in order to evaluate the presence of necrosis and neuronal deficits such as acidophilic and triangular shaped neurons, condensed and pyknotic nuclei, and vacuolization.

### 2.11. Behavioral Examination

At three days before surgery, the animals underwent daily training to assess neuromuscular and vestibulomotor functions. A total score was calculated for each rat. According to these tests, animals with higher scores had more deficits.

### 2.12. Neuromuscular Function

This test consisted of 6 subtests: forelimb flexion, twisting, resistance to lateral push, circling, hind limb placement, and inverted angle board gripping. The subtests and their scoring method have been carried out as described by Alexis et al. [34].

For forelimb flexion, a normal rat will extend both forelimbs toward the surface when it is held by the tail above a flat surface whereas infarcted animals will flex the paralytic forelimb. The degrees of flexion vary from mild wrist flexion and shoulder abduction to severe flexion of the entire forelimb. Torso twisting in a normal rat involves extension of the entire body toward the surface when held by the tail above a flat surface. The infected rats showed rotational behavior (body rotation) which was varied from mild twisting of the body to severe body movement which brings the head and forelimbs near the hind limbs. Twisting happens usually toward the injured and paralytic side. In the lateral push test the normal animal is held behind the shoulders and pushed to the left or right side. A normal rat shows equal resistance whereas the infarcted rat when pushed toward the contralateral side will show no resistance or a weaker resistance. During circling, the normal gate rats normally do not circle. However infarcted rats often circle toward the contralateral side. A normal rat during the hind limb placement test will immediately return its hind limb to the surface from which it has been removed; however infarcted rats show a delay in placement or no placement of the hind limb. In the inverted angle board test, the normal rat can be trained to turn 180 degrees and move to the top of the angled board. Infarcted rats cannot turn and move up the board.

### 2.13. Vestibulomotor Function (Beam Balance)

This test assesses cortical motor deficits. The test and its scoring method have been performed according to Alexis et al. [34] and Petullo et al. [35]. Briefly, the animal was positioned on a beam of 3/4 inches' width, 10 inches' length that was suspended 1 foot above a table top. A normal animal should maintain steady posture with all limbs on top of the beam for 60 s.

### 2.14. Statistical Analysis

Statistical analysis was performed using SPSS 17.0 software. The statistical differences between different groups were evaluated with one-way analysis of variance (ANOVA) followed by the Tukey test for multiple pairwise examinations. A value of *P* < 0.05 was considered statistically significant. According to the results of the Kolmogorov-Smirnov test, the data for behavioral assessments lacked normal distribution. Therefore the nonparametric Kruskal-Wallis and Mann–Whitney tests were used for statistical analyses.

## 3. Results

### 3.1. Immunohistochemistry Findings

**Figure 2:**
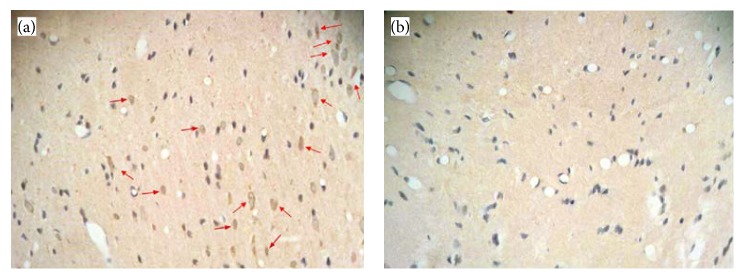
Immunohistochemical findings. (a) Treatment group. Intravenously transplanted bone marrow stromal cells 7 days after ischemia have migrated to the hippocampal area around the injured site. The arrows indicate Brdu-positive bone marrow stromal cells. (b) Vehicle group which received PBS 7 days after ischemia. There is no Brdu-positive bone marrow stromal cells.

Passage-3 BMSCs were transplanted into the rats. Three days before transplantation, the cells were labeled with Brdu (Sigma, Germany). After transcardial perfusion, we prepared 5 *μ*m paraffin sections in order to detect the BMSCs. The sections were stained with anti-Brdu antibody (Sigma, Germany) according to the immunohistochemistry kit instructions. Immunohistochemistry findings confirmed the presence and viability of transplanted cells at the area of lesion (hippocampus). The implanted BMSCs survived at the injury site (hippocampus). Light microscopy (Figure 2) showed that intravenously transplanted Brdu-positive BMSCs survived and migrated to the injured sites (CA1, CA3 areas of hippocampus).

### 3.2. TUNEL Staining

**Figure 3:**
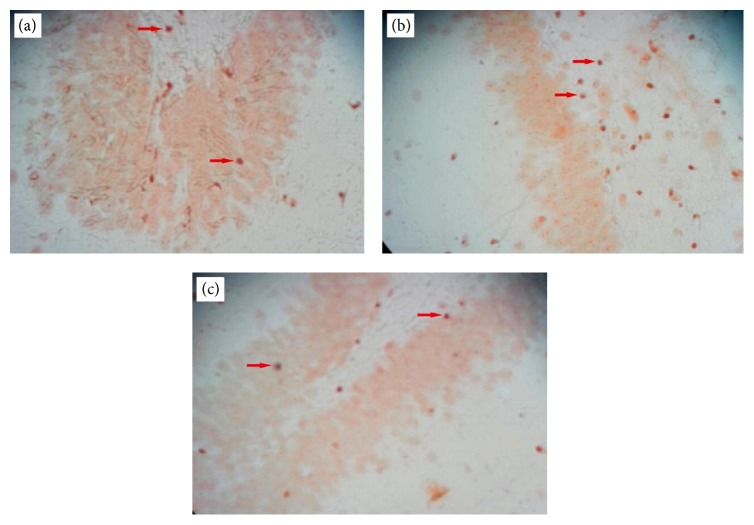
TUNEL reaction. (a) Control group with the lowest number of TUNEL positive (apoptotic) cells. (b) Ischemia group with the highest number of TUNEL positive (apoptotic) cells. (c) Treatment group with fewer TUNEL positive (apoptotic) cells compared to the ischemia group. The arrows show TUNEL positive cells (brown).

We used TUNEL staining to determine the number of apoptotic cells in the CA1 and CA3 areas of the hippocampus (Figure 3). The mean numbers of TUNEL positive cells were as follows: 7.05 ± 2.982 (control), 8.25 ± 2.693 (sham), 20.45 ± 10.308 (ischemia), 20.15 ± 9.67 (vehicle), and 10.0 ± 3.974 (treatment). The percent of TUNEL positive (apoptotic) cells were as follows: control (15.44%), ischemia (43.37%), treatment (23.61%), sham (17.92%), and vehicle (43.29%). The control group had the lowest and ischemia group had the highest numbers of apoptotic cells. The treatment group had fewer apoptotic cells than the ischemia group.

**Figure 4:**
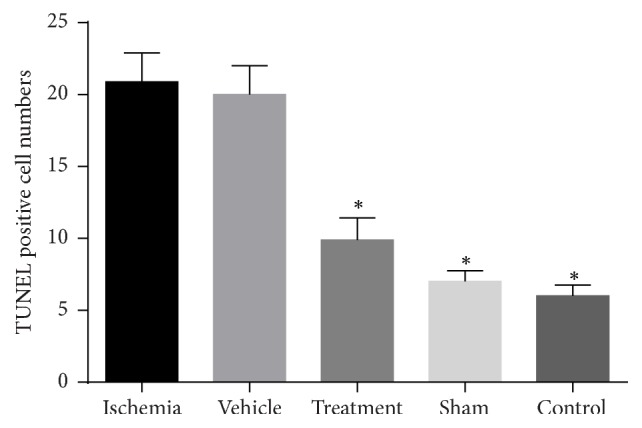
The mean number of TUNEL positive cells in the study groups. The ischemia group had the greatest number of TUNEL positive cells. There was a significant difference (shown with *∗*) in the numbers of apoptotic cells between the ischemia and control groups (*P* = 0.000).

A significant difference existed in the number of TUNEL positive cells between the control and ischemia groups (*P* = 0.000, Figure 4). There was a statistically significant difference in the number of TUNEL positive cells between the ischemia and treatment groups (*P* = 0.000, Figure 4).

### 3.3. Apoptotic Index Determination

The apoptotic index has been determined for each group as (1).

**Figure 5:**
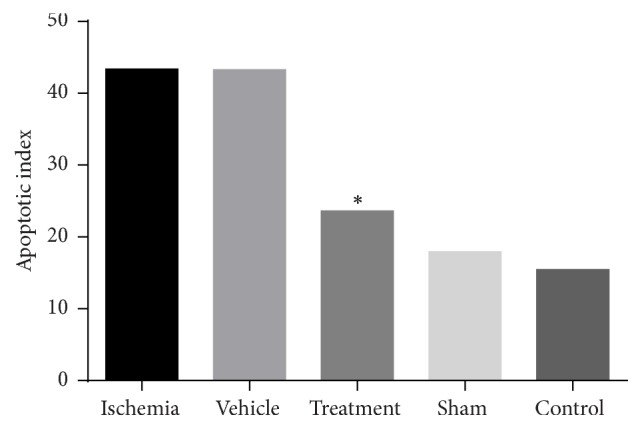
Apoptotic index in different groups. The results revealed that this index was less in the treatment group compared to the ischemia group (shown with *∗*).

In results, the apoptotic index was 43.37% (ischemia), 43.29% (vehicle), 23.61% (treatment), 17.92% (sham), and 15.44% (control). The apoptotic index in the treatment group was less than the ischemia group (Figure 5).

### 3.4. Histopathological Evaluation

**Figure 6:**
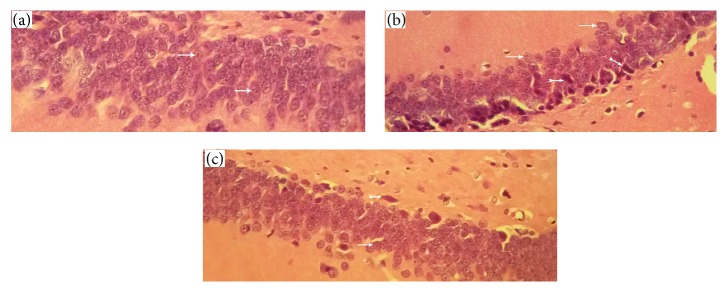
Histopathological assessment. (a) Control group neurons have an integrity and regular structure with normal morphology, round nuclei, prominent nucleolus, and clear cytoplasm. (b) Ischemia group neurons have numerous pyramidal cells with pyknotic nuclei and lack of nucleolus and hyperchromatic cytoplasm. (c) Treatment group have considerably decreased numbers of cells with pyknotic nuclei and lack of nucleolus and hyperchromatic cytoplasm compared to the ischemia group (thin arrows indicate normal cells and thick arrows indicate apoptotic cells).

Histopathological assessment showed that the control group neurons and neural tissue were intact and had normal morphology (Figure 6(a)). These cells had integrity and regular structure in the cerebral tissue. The pyramidal cells had round nuclei, prominent nucleolus, and clear cytoplasm. The ischemia group had numerous pyramidal cells with pyknotic nuclei, lack of nucleolus, and hyperchromatic cytoplasm compared to cells of the control group (Figure 6(b)). In the BMSC treatment group, we observed considerably decreased numbers of cells with pyknotic nuclei, lack of nucleolus, and hyperchromatic cytoplasm compared to the ischemia group (Figure 6(c)).

### 3.5. Behavioral Examination

#### 3.5.1. Effect of Bone Marrow Mesenchymal Stem Cells (BMSCs) on Neuromuscular Function

The neuromuscular function test consisted of 6 subtests: forelimb flexion, twisting, resistance to lateral push, circling, hind limb placement, and inverted angle board gripping. The details of these tests are explained in Section 2.12. In this study, we have graded the severity and degrees of neuromuscular function according to Table 1. Neuromuscular function test assessed neuromuscular cortical deficits. Animals with higher scores have more deficits, which means that higher scores indicate poor outcome and lower scores indicate favorable outcome.

**Figure 7:**
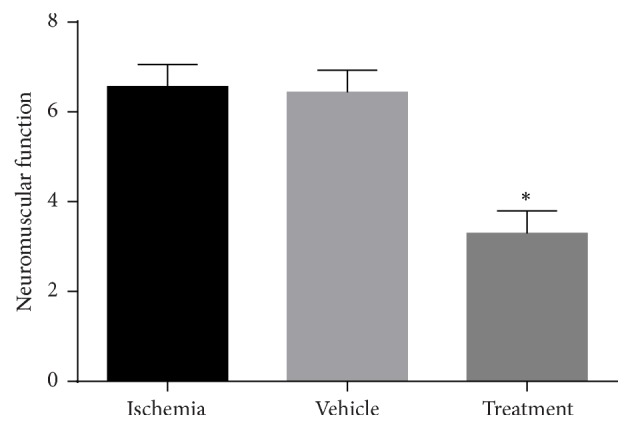
Effects of bone marrow mesenchymal stem cells (BMSCs) on neuromuscular function in rats after cerebral ischemia. Significant difference with treatment group (*P* < 0.05) (shown with *∗*).

In results, there was a significant increase (*P* < 0.001) in neuromuscular function test scores in the ischemia (6.56) and vehicle (6.43) groups compared to the control (0.0) group. The BMSC treatment group showed significant improvement (3.12) in neuromuscular function compared with the ischemia and vehicle groups (*P* < 0.001) (Figure 7).

#### 3.5.2. Effect of Bone Marrow Mesenchymal Stem Cells (BMSCs) on Vestibulomotor Function

The vestibulomotor function test assesses motor cortical deficits. Briefly the animals were positioned on a beam of 3/4 inches' width, 10 inches' length that was suspended 1 foot above a table top. A normal animal should maintain a steady posture with all limbs on top of the beam for 60 s. The severity and degrees of vestibulomotor function were graded according to Table 2. Animals with higher scores have more deficits; in other words, higher scores indicate poor outcome and lower scores indicate favorable outcome.

**Figure 8:**
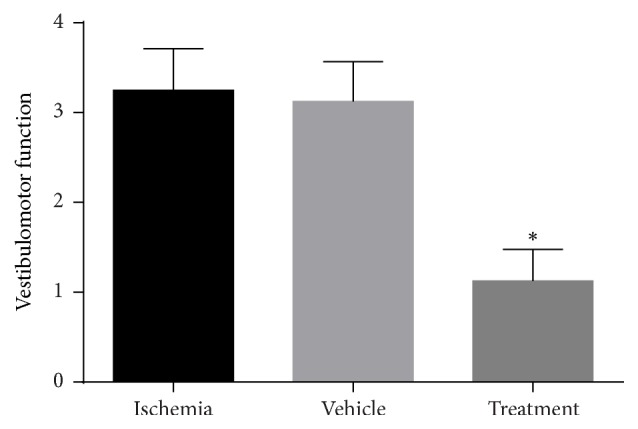
Effects of bone marrow mesenchymal stem cells (BMSCs) on vestibulomotor function in rats after cerebral ischemia. Significant difference with treatment group (*P* < 0.05) (shown with *∗*).

In this study there was a significant increase in vestibulomotor function score in the ischemia (3.25) and vehicle (3.00) groups (*P* < 0.001) compared with the control (0.0) group. The treatment group showed significant improvement (1.12) in vestibulomotor function compared with the ischemia group (*P* < 0.001, Figure 8).

## 4. Discussion

This study showed that intravenous transplantation of BMSCs one week following an ischemia-reperfusion injury decreased the apoptosis level of pyramidal neurons in the CA1 and CA3 areas of the hippocampus and improved functional recovery. According to similar studies in the field of cerebral ischemia, the treatment processes (especially with stem cells) are begun 7 days after ischemia induction. Researchers believe that, during the first week after cerebral ischemia, there is a collection of toxins and glutamate neurotransmitter and free radicals in the injured area of the brain and after 7 days these toxins are removed and the area is prepared to receive any new treatment (stem cells, growth factors, neurotrophins, etc.) [36, 37].

As is showed in the results, the intravenous transplantation of BMSCs one week following an ischemia-reperfusion injury decreased the apoptosis level of pyramidal neurons in the CA1 and CA3 areas of the hippocampus and improved functional recovery. In addition, the results of BMSCs culture (Figure 1) and labeling with Brdu immunohistochemistry showed that Brdu-positive BMSCs survived and migrated to the injury site (hippocampus) (Figure 2). Moreover, the results of TUNEL staining for determination of apoptotic cells following the ischemic injury (Figure 3) showed that the mean number of TUNEL positive cells in the ischemia group was the highest and the control group had the lowest number of TUNEL positive cells and there was a significant difference in the number of apoptotic cells between the ischemia and control groups (*P* = 0.000, Figure 4). The apoptotic index was lower in the treatment group compared to the ischemia group (Figure 5). Finally, these results indicated that BMSCs injection after ischemia-reperfusion led to survival of hippocampal cells and reduction of apoptosis in these cells.

The abovementioned findings indicated that the ischemia-reperfusion model in which the bilateral common carotid arteries were occluded for 30 min and then reperfused was a valid experimental model. The majority of apoptotic cells were observed in the ischemia group, which confirmed that this model could cause apoptosis in the highest number of hippocampal cells. A significant reduction was observed in the number of apoptotic cells in the treatment group compared to the ischemia group. Additionally, a statistically significant difference existed in the number of apoptotic cells between the treatment and ischemia groups (*P* = 0.000).

In histopathological assessment results it is clear that the control group neurons had normal morphology, round nuclei, prominent nucleolus, and clear cytoplasm, while the ischemia group had many pyramidal cells with pyknotic nuclei, lack of nucleolus, and hyperchromatic cytoplasm. The BMSC treatment group had a significantly decreased number of cells with pyknotic nuclei, lack of nucleolus, and hyperchromatic cytoplasm compared to the ischemia group (Figures 6(a)–6(c)). These findings indicated that administration of BMSCs after ischemia-reperfusion reduced the number of apoptotic cells.

The results of behavioral examination for evaluating the sensorimotor function in the three areas, neuromuscular, vestibulomotor, and complex neuromotor, displayed a decrease in the neuromuscular function score in the BMSC treatment group compared to the ischemia group. This result indicated a significant improvement (*P* < 0.001) in neuromuscular function in the treatment group. In addition, the results of vestibulomotor function and complex neuromotor function showed a decrease in scores of these tests in the treatment group compared to the ischemia group. These outcomes point out the significant improvement (*P* < 0.01) in vestibulomotor function and complex neuromotor function in the treatment group. Finally, it can be concluded in behavioral examinations that the intravenous injection of BMSCs after ischemia-reperfusion significantly improved functional recovery.

A number of researchers believe that BMSCs secrete numerous growth factors [38], neurotrophic factors [39], and brain natriuretic peptides which reduce neuronal apoptosis [40]. On the other hand, some scientists explain that BMSCs are multipotent cells capable of differentiating into neurons and glial cells in vitro [41] and in vivo [42]; hence these neurons and glial cells can replace damaged cells [43], regenerate injured tissue, and promote functional recovery.

Mahmood et al. studied an experimental model of traumatic brain injury. They injected BMSCs intravenously and reported that these cells migrated to the animals' brains and improved functional recovery [44]. They measured nerve growth factor (NGF), brain derived neurotrophic factor (BDNF), and fibroblast growth factor *β* (bFGF) and concluded that these factors might improve functional recovery [45]. Crigler et al. reported that injected BMSCs secreted neurotrophins, growth factors, and cytokines, which caused cell proliferation, survival, and differentiation [46]. Chen et al. demonstrated that BMSCs secreted NGF, BDNF, glial cell derived neurotrophic factor (GDNF), neurotrophin 3 (NT3), bFGF, vascular endothelial growth factor (VEGF), hepatocyte growth factor (HGF), and ciliary neurotrophic factor (CNTF) [47].

Nicaise et al. transplanted BMSCs in an experimental amyotrophic lateral sclerosis (ALS) model. Their study showed that BMSCs expressed NGF, FGF2, and insulin-like growth factor (IGF). They concluded that BMSCs with expression of growth factors caused neurons to survive in the brain [48]. Joghataei et al. cultured BMSCs and Schwann cells (SCs) and then transplanted them into an experimental model of spinal cord injury. Findings of their study showed that these cells promoted functional recovery and caused axonal regeneration in the injured animals. They proposed that BMSCs and SCs secreted neurotrophic factors which resulted in functional recovery in these animals [49]. Munoz et al. have reported that transplantation of BMSCs led to increased neurogenesis in hippocampal cells [50]. Chen et al. developed an experimental stroke model by occluding the middle cerebral arteries. Following intravenous injection of BMSCs, they evaluated the level of neurogenesis, apoptosis, and bFGF expression. Their findings showed that BMSCs increased both neurogenesis and bFGF expression and decreased apoptosis [51].

All of the abovementioned findings agree with the results of the current study. We demonstrated that intravenous injection of BMSCs in an experimental model of ischemia-reperfusion led to hippocampal cell survival, reduction of apoptosis in these cells, and improvement of functional recovery. It has been proposed that post-ischemia-reperfusion injected BMSCs express neurotrophic and growth factors which prevent neuronal apoptosis, resulting in neuron and glial cell survival, which lead to enhanced behavioral recovery. These factors have provided a suitable environment that promoted axonal growth and resulted in locomotor recovery. BMSCs can improve vascularization through the expression of VEGF leading to damaged neural tissue repair [52].

Our findings have suggested that administration of BMSCs one week after ischemia-reperfusion can reduce hippocampal cell apoptosis in the rat model. This has resulted in improved locomotor function and it may be employed as a useful method for treatment of human cerebral ischemia. The BMSCs have a number of advantages compared to other cells commonly used in cell therapy; they can be easily isolated and cultured and their transplantation is safe without immunological reactions. Indeed, further studies are required for the development of this method as a treatment for strokes in humans.

## 5. Conclusion

In this study, the results of intravenous administration of BMSCs in an animal model of ischemia-reperfusion revealed considerable reduction of apoptosis in hippocampal cells, along with neuron survival and functional recovery. This may be a beneficial treatment method for ischemic injuries. It has been proposed that post-ischemia-reperfusion injected BMSCs express neurotrophic and growth factors which prevent neuronal apoptosis, resulting in neuron and glial cell survival, which lead to enhanced behavioral recovery. These factors have provided a suitable environment that promoted axonal growth and resulted in locomotor recovery.

## Figures and Tables

**Table 1:** Clinical scoring criteria (neuromuscular function).

Criteria	Score	Description
Forelimb flexion	0.0	No flexion
	0.5	Mild
	1.0	Moderate to severe

Torso twisting	0.0	No flexion
	0.5	Mild
	1.0	Moderate to severe

Lateral push	0.0	Equal resistances
	0.5	Weak resistance
	1.0	No resistance

Hindlimb placement	0.0	Immediately replaces
	0.5	Delay in replacing
	1.0	No replacement

Forelimb placement	0.0	Immediate replacement
	0.5	Delay in replacing
	1.0	No replacement

Inclined board	0.0	Turning 180 and then replacement
	0.5	Able to stay up but not go to top
	1.0	Tries to stay up but eventually slides down
	1.5	Tries to stay up but immediately slides down
	2.0	Not able to hold body up at all, immediately

Mobility	0.0	Normal
	1.0	Spontaneous movement to move
	1.5	Needs stimulus to move
	2.0	Unable to walk

Maximum score	9.0	

**Table 2:** Clinicl scoring criteria.

Vestibular function	Score	Description
Balance beam	0.0	Balances with all 4 paws on top of the beam
	1.0	Puts paws on side of beam or wavers
	2.0	1 or 2 limbs slip off beam
	3.0	3 limbs slip off beam
	4.0	Attempts to balance but falls off
	5.0	Animal drapes on beam then falls
	6.0	Falls without attempting to balance

Maximum score	6.0	

## Abbreviations

BMSCs: Bone marrow mesenchymal stem cells
Brdu: Bromodeoxyuridine
ROS: Reactive oxygen species
PBS: Phosphate-buffered saline
TUNEL: Terminal deoxynucleotidyl transferase-mediated dUTP end labeling
NGF: Nerve growth factor
BDNF: Brain derived neurotrophic factor
bFGF: Fibroblast growth factor *β*
GDNF: Glial cell derived neurotrophic factor
NT3: Neurotrophin 3
VEGF: Vascular endothelial growth factor
HGF: Hepatocyte growth factor
CNTF: Ciliary neurotrophic factor
ALS: Amyotrophic lateral sclerosis.
